# Acupotomy versus nonsteroidal anti-inflammatory drugs for knee osteoarthritis: Protocol for a systematic review and meta-analysis

**DOI:** 10.1097/MD.0000000000017051

**Published:** 2019-09-06

**Authors:** Renpan Zhang, Lixiang Li, Bin Chen, Hong Liu, Jing Liu, Liangzhi Zhang, Zhongbiao Xiu

**Affiliations:** a The Affiliated People's Hospital of Fujian University of Traditional Chinese Medicine, Fuzhou 350004; b Fujian University of Traditional Chinese Medicine, Fuzhou 350122, Fujian Province, China.

**Keywords:** acupotomy, knee osteoarthritis, meta-analysis, NSAIDs, protocol, systematic review

## Abstract

**Background:**

Knee osteoarthritis (KOA), a common clinical chronic osteoarthropathy, has adverse effects on the working ability and life quality of patients. At present, acupotomy and nonsteroidal anti-inflammatory drugs (NSAIDs) have been widely applied on KOA in China and has achieved satisfactory results. However, there is no systematic review comparing the effectiveness of these two therapies for KOA. Therefore, this study will assess the efficacy and safety of acupotomy in treating KOA.

**Methods and analysis:**

Several databases including CNKI, Wanfang Database, CBM, VIP, PubMed, Cochrane library, Web of Science, and Embase will be used by two independent researchers to search the related clinical RCTs about acupotomy therapy for KOA patients until May 2019. Subsequently, meta-analysis will be conducted by using Review Manager 5.3, and the related data will be analyzed by using the method for GRADE. The continuous data will be presented as the WMD or SMD with 95% CI, while dichotomous data will be shown as the RR with 95% CI.

**Conclusion:**

Our results review will provide evidence to determine whether acupotomy can achieve the effect of NSAIDs, or whether it has advantages and safety compared with NSAIDs for patients with KOA.

**Ethics and dissemination:**

This study will not involve personal information. The ethical approval will not be required. This systematic review will be disseminated electronically through a peer-reviewed journal or international conference presentations.

**PROSPERO registration number:**

CRD42019129089.

## Abstract 2

Supplemental Digital Content is available in the text

**Strengths and limitations**

- At present, there is no systematic review comparing the effectiveness and safety between acupotomy and NSAIDs for KOA. Our systematic assessment will help clinicians to determine the optimal treatment options for KOA.
- One limitation of this systematic review is that different types of acupotomy therapies may lead to heterogeneity.
- Another limitation is that only the studies published in English or Chinese will be included due to the language barriers, thus causing language bias.

## 1 Introduction

Knee osteoarthritis (KOA), a kind of most common joint disorder among the elderly, is characterized by arthralgia, ankylosis and functional disorders. At present, KOA is considered as a common health problem all over the world. In the United States, approximately 14 million Americans suffered from this disease.^[1]^ An epidemiological survey in China showed that the prevalence of KOA was 18% and that ranged from 9% to 13% for men and from 16% to 23% for women.^[2]^ According to relative statistics, the incidence of this disease in Sweden was estimated approximately 15.4% among the adults aged from 56 to 84 years.^[3]^ Moreover, with the societies aging in the whole world, the prevalence of osteoarthritis continues to rise, which further leads to a reduction in life quality and wastes valuable medical resources.^[4]^

The aim of KOA management is to alleviate the symptoms of stiffness and ache.^[5]^ Some traditional therapies including nonsteroidal anti-inflammatory drugs (NSAIDs),^[6]^ intra-articular injections^[7]^ and paracetamol.^[8]^ NSAIDs are one of the effective methods recommended by clinical guidelines,^[9]^ which play a role in inhibiting inflammation and relieving pain. For patients with mild KOA, topical NSAIDs are recommended, while, for patients with moderate to severe KOA, NSAIDs are recommended to be administered orally, to control symptoms at the minimum local analgesic dose. However, oral NSAIDs are often concomitant with severe gastrointestinal reactions and other side effects, leading a restriction of NSAIDs in the clinical application.^[10]^ Due to these disadvantages, more and more patients suffering from KOA are turning to alternative or complementary therapies. In clinical practice, the acupotomy therapy has been widely used in China to treat pain in patients with various diseases.^[11–14]^

Acupotomy consists of flat head and cylindrical body. Due to the special structure, it could be used to cut and strip local lesion tissues.^[12]^ At present, acupotomy therapy is considered as a combination of Traditional Chinese Medicine (TCM) and modern surgery. In TCM theory, acupotomy is thought to promote Qi-blood circulation. In Western medicine, the action mechanism of acupotomy still remains unclear. However, accumulated evidences have shown that acupotomy could release adhesion, adjust the mechanical balance between the knee joint ligaments, speed up and improve lymphatic circulation, and loosen abnormal tissue pressure.^[15–17]^ Some studies in animals and humans signified that acupotomy could also inhibit the expressions of pro-inflammatory cytokines, promote the synthesis of cartilage cell metabolism to some extent, and repair the damage of cartilage.^[17,18]^

Previous clinical trials compared acupotomy with NSAIDs in terms of their KOA treatment effect.^[19,20]^ However, no identical conclusions were obtained. Thus, this review is to explore whether acupotomy therapy has the same therapeutic effect as that of NSAIDs, or whether it is more effective and safer when comparing with NSAIDs. We firmly believe that this study will provide convincing evidence by using these above strict search strategy and outcome evaluation.

## 2 Methods

### 2.1 Inclusion criteria for study selection

#### 2.1.1 Type of study

Included studies: The RCTs about acupotomy versus NSAIDs for KOA will be included in this study, but Quasi-RCTs and randomized crossover studies will be excluded. In addition, the studies involving “randomization” will also be included, and they will be defined as “the risk of bias assessment” if the detailed description about this randomization process fails to be provided. On the contrary, those studies using incorrect randomization methods including alternate distribution will be excluded. Due to the characteristics of acupotomy therapy, it will not cause blinding. Additionally, all the included studies are published in Chinese and English.

The exclusion criteria are listed: firstly, replicated studies; secondly, no specific diagnosis criterions for KOA; thirdly, reviews and theory researches; and finally, animal experiments.

#### 2.1.2 Types of participants

Whether the subjects are suffered with skeletal osteoarthritis is not related to gender, ethnic or educational backgrounds and economic ability, and meet the following criteria: firstly, The American College of Rheumatism/NICE guide: definition, classification and diagnosis of KOA.^[21,22]^ secondly, Chinese guidelines for osteoarthritis diagnosis and treatment.^[5]^

If the specific diagnosis criteria are not recorded in the study, the diagnosis for KOA must be based on identifiable important features including age 45–75 years, chronic knee pain for the last 6 months and morning stiffness shorter than 30 minutes.^[23]^

#### 2.1.3 Types of interventions

Experimental interventions: The studies in which the treatment group receiving acupotomy therapy will be included. No restrictions are imposed on times of treatment, frequency of treatment, and length of treatment period.

Comparator interventions: Patients in the control group will be administered NSAIDs alone. NSAIDs (topical or oral) include traditional (tNSAIDs) and selective cyclooxygenase-2 inhibitors (COXIBs). Other alternative therapeutic interventions (e.g., Chinese herbal medicine, usual acupuncture, moxibustion) will be excluded.

The procedure of acupotomy was reported in full compliance with the standardized reporting methods, such as the Standard of the Basic Manipulations of Acupotomy (ZJ/T D001-2014).

#### 2.1.4 Types of outcome measures

Primary outcomes: firstly, study data will include some scales that measure pain intensity as a result, such as visual analogue scale (VAS) and numerical rating scale (NRS). Secondly, Western Ontario and McMaster Universities osteoarthritis index (WOMAC).

Secondary outcomes: firstly, the overall evaluation of proportions of improved or cured patients. Secondly, the MOS 36-item short-form health survey (SF-36). Thirdly, the incidences of adverse events.

### 2.2 Data sources and search strategy

Eight electronic bibliographic databases including Web of Science, PubMed, Embase, Cochrane library, China Biology Medicine disc (CBM), China National Knowledge Infrastructure (CNKI), Chinese Scientific Journal Database (VIP), and Wanfang will be independently searched by two researchers from inception to May 2019. The studies published in English and Chinese will be included. The search strategy is established based on the Cochrane handbook guidelines. The PubMed search strategy will be presented in Appendix 1. And other searches will be conducted based on these search results.

Medical journal lists will be manually searched as a supplement, such as: *Chinese Acupuncture and Moxibustion* (1981–May 2019), *Acupuncture Research* (1976–May 2019), and *Journal of Traditional Chinese Medicine* (1960–May 2019). In addition, other potential literatures will also be searched in OpenGrey.eu.

### 2.3 Data collection and analysis

#### 2.3.1 Selection of studies

**Figure 1:**
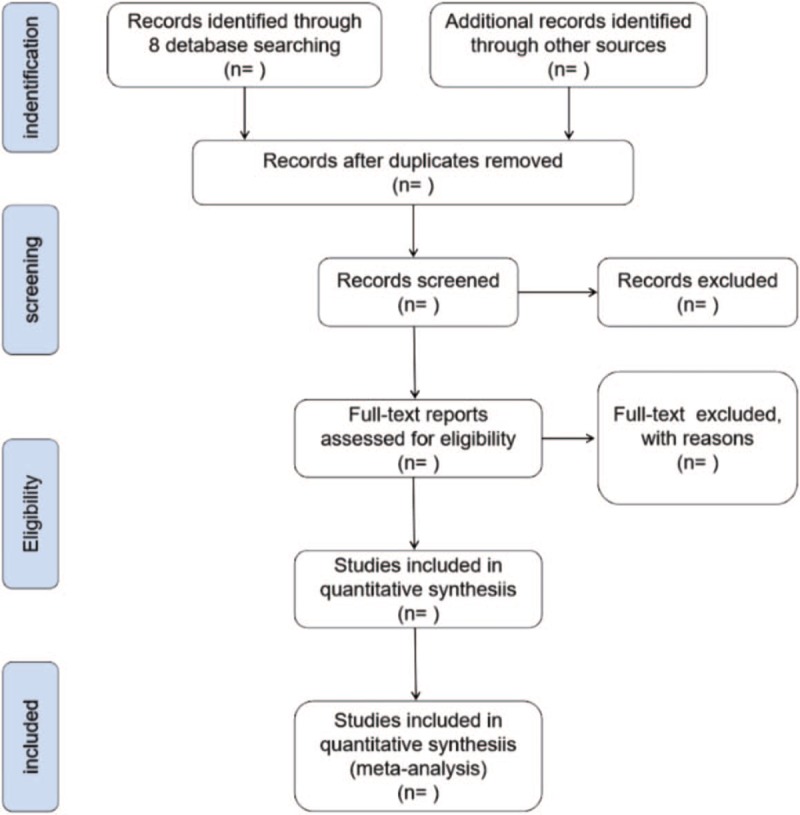
PRISMA flow diagram of study process.

All searched literatures will be imported into EndNote library, and the repeated references will be removed. Two review authors (Jing Liu and Liangzhi Zhang) will independently review the titles, abstracts and full text (all possible relevant articles) for further evaluation after receiving training to develop a common understanding for the screening criteria and later procedures. Any disagreements will be eliminated via discussion between these two authors, or through consulting another co-author (Zhongbiao Xiu). The selection procedure is illustrated in Figure 1.

#### 2.3.2 Data extraction and management

Bin Chen and Hong Liu will independently extract data from the included trials by using a data extraction form. The related data include: general information (such as title, authors, year, and published country), details of study (such as design, inclusion and exclusion criteria, blinding, randomization, and sample size), subject information (such as age and numbers), procedures of intervention (such as type of acupotomy therapy, acupoints, treatment details, frequency, and NSAIDs drugs), types of outcomes (such as primary, secondary, and safety outcomes), and other detailed information. If necessary, the contact with corresponding authors from the included trials can be established to obtain further information.

#### 2.3.3 Evaluation of bias risk

Jing Liu and Liangzhi Zhang will assess the bias risk of all the included studies by using the Cochrane Collaboration's tool.^[24]^ This tool contains seven options: random sequence generation, allocation concealment, blinding of participants and personnel, blinding of outcome assessors, completeness of outcome data, selective outcome reporting, and other biases. The bias risks will be classified as low, unclear and high. The results will be shown as the risks of bias graph and bias summary using the Cochrane Collaboration's software (RevMan version 5.3). If there are any disagreements, the arbiter (Zhongbiao Xiu) will do the final judge.

#### 2.3.4 Detection of therapy effect

Continuous data will be expressed as the mean difference (MD) with 95% CI, while dichotomous data will be shown as RR with 95% CI.^[25]^ When there is the same outcome measured in different ways, the SMD with 95% CI will be used to express the intervention effect.

#### 2.3.5 Dealing with missing data

If some necessary data are missing, the contact with corresponding authors from the included studies will be established to obtain the missing or incomplete data by e-mail. If failing to obtain the missing data, the existing data will be analyzed, and a sensitivity analysis will be performed to eliminate the potential effects caused by the missing data.

#### 2.3.6 Evaluation of heterogeneity

Chi-square test and *I*^2^ value will be used to assess the heterogeneity in our meta-analysis. *P* < .10 or *I*^2^ value more than 50% will be regarded as significance based on the Cochrane Handbook guideline. For substantial heterogeneity, possible reasons will be investigated through sensitivity and subgroup analyses, or only descriptive analysis can be performed.

#### 2.3.7 Assessment of reporting bias

If there are more than 10 included studies in our meta-analysis, the visual asymmetry on the funnel plots will be applied for the assessment of potential reporting biases. Also, we will perform Egger's test to assess plots visually.

#### 2.3.8 Data synthesis

This meta-analysis will be performed by using the Review Manager version 5.3. When there is a low statistical heterogeneity among the outcomes, the fixed-effect model will be used. When there is normal statistical heterogeneity among the outcomes, its source will be further investigated. After removing the impact of obvious clinical heterogeneity, the random-effect model will be employed.

#### 2.3.9 Subgroup analysis

No pre-subgroup plan will be conducted in this meta-analysis. The subgroup analysis can also be conducted according to the various interventions, controls and different outcomes if the data are available.

#### 2.3.10 Sensitivity analysis

If there are adequate studies (no less than three studies), the sensitivity analysis for primary outcomes will be performed to detect the robustness of outcomes. In brief, this analysis can be repeated after excluding the influence of the studies with low quality, supplementing the missing data or selecting different statistical models.

#### 2.3.11 Assessment of outcome quality

The Grading of Recommendations Assessment, Development, and Evaluation (GRADE) will be performed to assess the quality of main outcomes. Bias risk, consistency, directness, precision, publication bias and other points will be assessed, and the assessments will be subsequently categorized into four grades: very low, low, moderate, and high.

## 3 Discussion

Acupotomy therapy for KOA has lots of advantages since it could turn open surgery into a miniature surgery with higher acceptability and less pain, thereby decreasing surgical time, risks and costs. Although many clinical studies confirmed that acupotomy was effective for alleviating the symptoms of KOA, there is no comprehensive systematic review comparing the effectiveness of these two therapies for KOA. We hope this study will provide the latest available evidence to demonstrate whether there are definitive advantages in acupotomy therapy relative to NSAIDs in the patients diagnosed as KOA. Of course, there are several limitations in this study. Firstly, different types of acupotomy therapies and methodology qualities may lead to heterogeneity. Secondly, due to the language barriers, only the studies reported in English or Chinese will be included. Finally, KOA severity and different measurement tools may also bias the outcome.

### 3.1 Publication plan

This systematic review will be published in a peer-reviewed journal and will be disseminated electronically and in print.

## Author contributions

**Conceptualization:** Renpan Zhang, Zhongbiao Xiu.

**Formal analysis:** Renpan Zhang, Lixiang Li.

**Methodology:** Renpan Zhang, Lixiang Li, Bin Chen, Hong Liu, Jing Liu, Liangzhi Zhang.

**Software:** Renpan Zhang.

**Validation:** Zhongbiao Xiu.

**Writing – original draft:** Renpan Zhang, Lixiang Li.

**Writing – review & editing:** Renpan Zhang, Zhongbiao Xiu.

## Supplementary Material

Supplemental Digital Content
