# Supplementary material for: Acupotomy versus nonsteroidal anti-inflammatory drugs for knee osteoarthritis: Protocol for a systematic review and meta-analysis
Source: Medicine (Baltimore). 2019 Sep 6;98(36):e17051. doi: 10.1097/MD.0000000000017051 (PMC6739018; doi:10.1097/MD.0000000000017051)
Supplement: Supplemental Digital Content [file medi-98-e17051-s001.docx]

**Additional file 1. PubMed search strategy**

#1 “randomized controlled trial” [pt] OR “controlled clinical trial” [pt] OR “randomized” [ti, ab] OR “placebo” [ti, ab] OR “trial” [ti, ab] OR “groups” [ti, ab];

#2 clinical trials as topic [mesh: noexp];

#3 #1 OR #2;

#4 “acupotomy” [ti, ab] OR “needle scalpel” [ti, ab] OR “needle knife” [ti, ab] OR “acupotomlogy” [ti, ab] OR “miniscalpel acupuncture” [ti, ab] OR “miniscalpel needle” [ti, ab] OR “stiletto needle” [ti, ab] OR “Xiaozhendao” [ti, ab];

#5 “Osteoarthritis, Knee” [Mesh] OR “Knee Osteoarthritides” [ti, ab] OR “Knee Osteoarthritis” [ti, ab] OR “Osteoarthritides, Knee” [ti, ab] OR “Osteoarthritis Of Knee” [ti, ab] OR “Knee, Osteoarthritis Of” [ti, ab] OR “Knees, Osteoarthritis Of” [ti, ab];

#6 “Antiinflammatory Agents, Non Steroidal” [Mesh] OR “NSAIDs” [ti, ab] OR “Non-Steroidal Anti-Inflammatory Agents” [ti, ab] OR “Anti Inflammatory Agents, Nonsteroidal” [ti, ab] OR “Nonsteroidal Antiinflammatory Agents” [ti, ab] OR “Analgesics, Anti-Inflammatory” [ti, ab] OR “Anti-Inflammatory Analgesics” [ti, ab] OR “Aspirin-Like Agents” [ti, ab];

#7 #3 AND #4 AND #5 AND #6;

The same search items will be used to search all electronic databases.
